# Development of multifunctional nanocomposites for controlled drug delivery and hyperthermia

**DOI:** 10.1038/s41598-021-84927-x

**Published:** 2021-03-09

**Authors:** Vladimir Hovhannisyan, Katarina Siposova, Andrey Musatov, Shean-Jen Chen

**Affiliations:** 1 College of Photonics, National Chiao Tung University, Tainan, 711 Taiwan; 2 Department of Biophysics, Institute of Experimental Physics, Slovak Academy of Sciences, Watsonova 47, 04001 Kosice, Slovakia

**Keywords:** Neuroscience, Nanoscience and technology, Optics and photonics

## Abstract

Magnetic nano/micro-particles based on clinoptilolite-type of natural zeolite (CZ) were fabricated and were expected to act as carriers for controlled drug delivery/release, imaging and local heating in biological systems. Adsorption of rhodamine B, sulfonated aluminum phthalocyanine and hypericin by magnetic CZ nano/micro-particles was investigated, as was the release of hypericin. Using an alternating magnetic field, local temperature increase by 10 °C in animal tissue with injected magnetic CZ particles was demonstrated. In addition, the CZ-based particles have been found to exhibit an anti-amyloidogenic effect on the amyloid aggregation of insulin and lysozyme in a dose- and temperature-dependent manner. Therefore, the mesoporous structure of CZ particles provided a unique platform for preparation of multifunctional magnetic and optical probes suitable for optical imaging, MRI, thermo- and phototherapy and as effective containers for controlled drug delivery. We concluded that magnetic CZ nano/micro-particles could be evaluated for further application in cancer hyperthermia therapy and as anti-amyloidogenic agents.

## Introduction

The development of effective methods and materials for targeted transport, controlled release of bioactive substances and diagnostic probes continues to be an important area of modern pharmaceutics. Efficacious drug deposition in the target site and operated activation of therapeutic and imaging agents reduce treatment time, while avoiding adverse damage and side effects resulting from systemic administration. Currently, various types of magnetic nano- and microcapsules are synthesized for magnetically assisted delivery, controlled drug release, hyperthermia and magnetic resonance imaging (MRI)^1–5^. For biomedical applications, silica and ceramic host matrices are successfully used to develop biocompatible magnetic nanocomposites^3,6^. In particular, silica shells can prevent iron against oxidation for at least 6 months in air and 1 month in aqueous solution. In addition, these magnetic devices are successfully applied for loading and delivery of anticancer drugs in biological systems^3^. Another prospective porous material is zeolite, which shares many of the favorable properties of silica, especially in biomedical applications related to transportation of low molecular weight drugs and imaging agents. Thus, zeolites are increasingly seen as very promising containers also for drug delivery. Zeolites are naturally occurring or synthetic micro-mesoporous crystalline aluminosilicates. The crystalline structure of zeolites composed of a 3D tetrahedral framework of oxygen-sharing AlO_4_ and SiO_4_ groups. The most widespread and studied type of zeolite among more than 40 types of natural and 200 types of synthetic zeolites, is natural zeolite named clinoptilolite (CZ). CZ has a negative charge, large specific surface area (10–25 m^2^/g) and high resistance to extreme temperatures^7,8^. The mineral pore size (PS) distribution can be characterized by two narrow (PS1 < 2 nm, 2 < PS2 < 5 nm) and one broad (PS3 > 5 nm) curves. The primary porosity with PS1 < 2 nm is caused by the specific crystal building of CZ grains and the system of meso- and macropores (secondary porosity, with PS2 and PS3) is related to sizes and structural features of CZ mineral grains in the rock.

Pharmacological and toxicological studies demonstrate that CZ is not harmful to living cells, it is inert, non-toxic and safe for use in human and veterinary medicine^9–11^. Clinically formulated different CZ products are commercially available in Europe and the USA^12^. The successful use of original and modified forms of CZ in agriculture as a food supplement and in biomedicine as an excellent antioxidant, detoxifying, gastric antacid, anti-diarrheic, anti-hyperglycemic, antiviral and anti-inflammatory agent has been described in numerous publications^10,11,13^. In addition, the role of CZ as a novel matrix for the delivery and slow-release of ions, organic molecules and drugs has been proposed^14,15^. Furthermore, the protective effect of modified CZ against oxidative stress and plaque generation in transgenic mice models of Alzheimer disease is demonstrated and CZ is proposed as a potential preventive agent in Alzheimer’s disease and other neurological disorders^16^.

The optical properties of natural CZ have been studied and the characteristic CZ emission spectrum in 350–600 nm spectral range with excitation spectrum in 220–400 nm range and a maximum position around 280 nm is observed. It is demonstrated that CZ luminescence is associated with local defects and imperfections in crystals^17^. Recently, multiphoton imaging of CZ nano/micro-particles has been performed, and adsorption and release of photodynamic dyes by CZ particles using two-photon excited luminescence and second harmonic generation imaging have been quantitatively studied^18^.

The presences of several types of porosity and also the possibility to modify physicochemical properties of the surface through functionalization by various moieties are important features of zeolites. Both synthetic and natural zeolites are capable to effectively bind magnetic materials and zeolite magnetic particles fabricated in this way have strong magnetic properties, which allow to manipulate them, separate from other materials and use for adsorption of contaminants and simple magnetic separation of the adsorbent from the medium^19,20^. Magnetic composites based on natural zeolites are considered to be biocompatible medical devices, and are used for efficient loading and slow release of drugs and fluorescent markers. In addition, magnetic zeolites are able to deliver the specific agents to the disease zone very efficiently and purposefully, which provides a significant improvement in medical diagnostics and treatments. Recently, various types of magnetic zeolite nanocomposites have been synthesized, and their suitability for loading anti-cancer chemotherapeutic drugs such as doxorubicin has been studied^21^.

Here we present the new results on application of magnetic clinoptilolite zeolite (MCZ) for adsorption and release of photodynamic dyes, hyperthermia and disaggregation/inhibition of amyloid fibrils in vitro.

## Results

### Scanning electron microscopy and multiphoton microscopy of CZ particles

**Figure 1:**
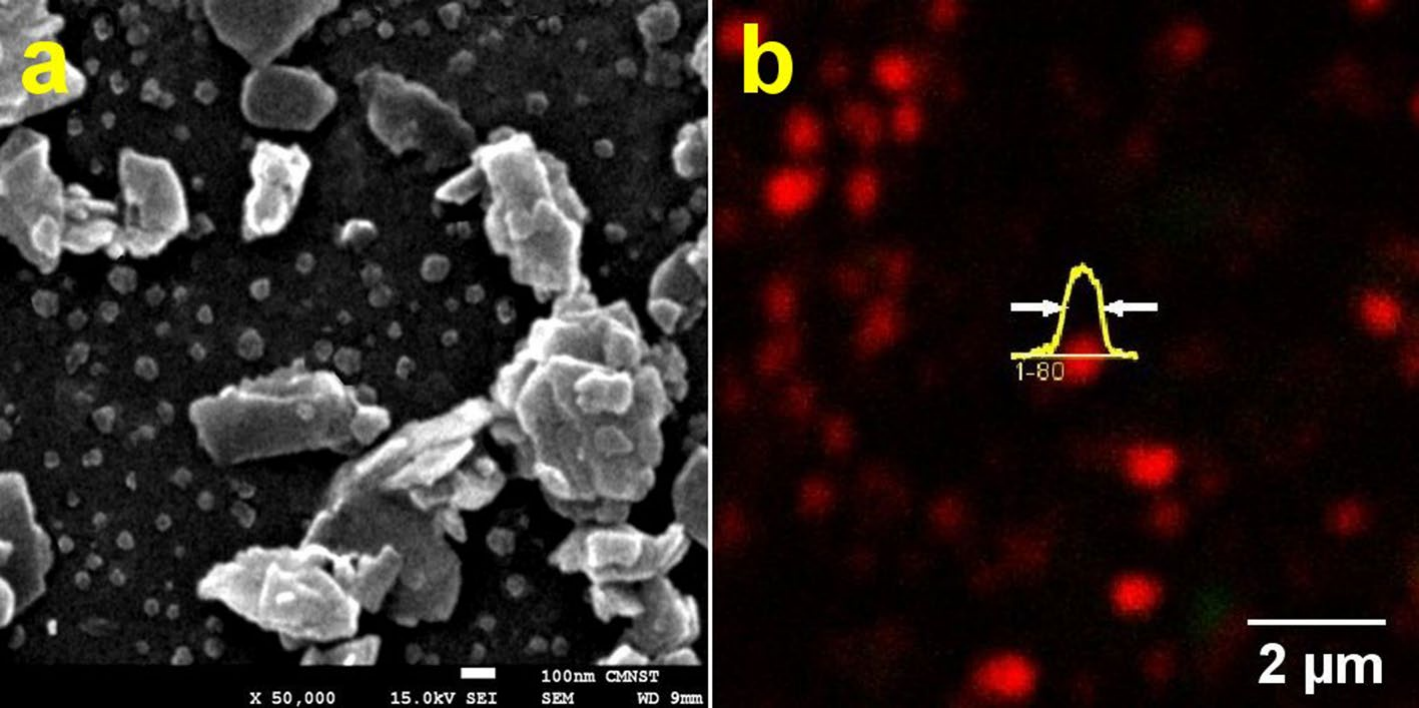
SEM (**a**) and SHG images (**b**) of CZ nanoparticles. The intensity profile of the individual nanoparticle along the center is shown in yellow. Scale bars represent 100 nm (**a**) and 2 µm (**b**). Excitation wavelength: 780 nm, objective: 40×/NA 1.2.

Sizes and morphology of CZ nanoparticles have been examined by scanning electron microscopy (SEM) (Fig. 1a). Nanometric CZ particles can be divided into 2 fractions. The first fraction consists of individual particles with characteristic sizes of 75 ± 20 nm, and the second fraction consists of clusters of 2 or more tablet-shaped particles that merge along their bases into the aggregation process. Most of the relatively large tablet-shaped particles in aggregates have a thickness of about 60–80 nm, and the average dimensions of the bases are 120 nm in width and 350 nm in length. These large particles can be separated from small particles by gravity. CZ particles and their interaction with dyes have been analyzed by multiphoton imaging (Fig. 1b). Natural CZ particles produce only second harmonic generation (SHG) signal at 780 nm fs excitation (red pseudocolor in Fig. 1b). When dye molecules are adsorbed by CZ particles, this leads to SHG quenching, and individual CZ particles emit only two-photon excited fluorescence^18^.

### Magnetic zeolite for drug adsorption and release

**Figure 2:**
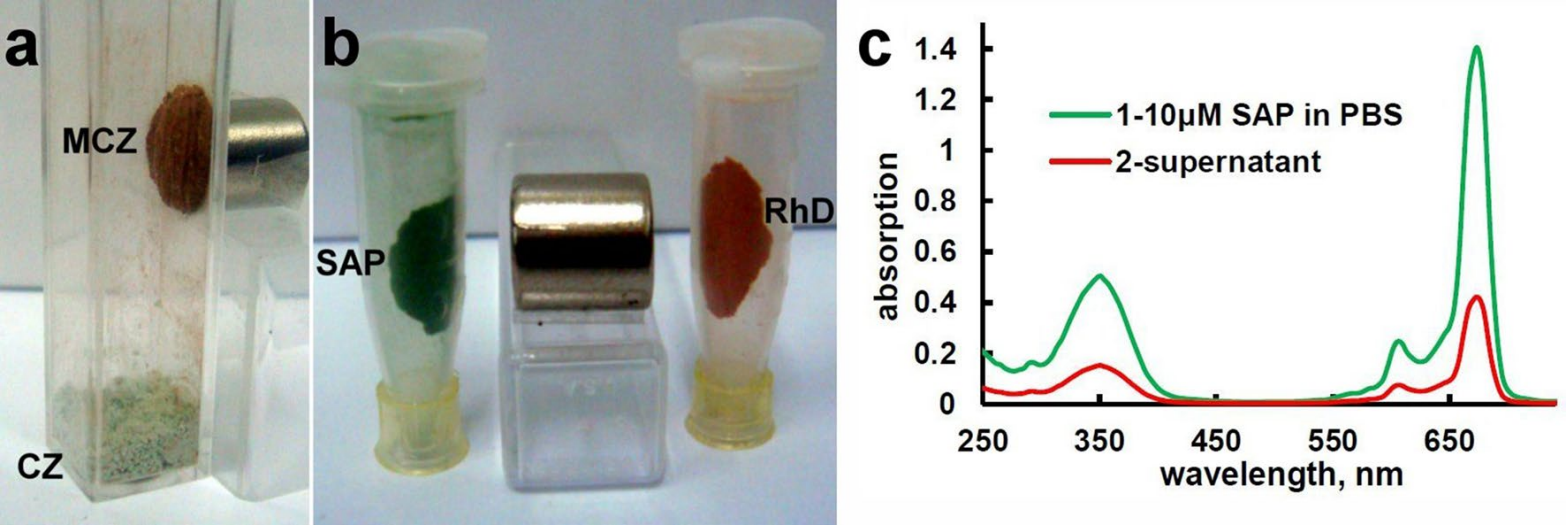
Magnetic separation of pure MCZ particles and MCZ with adsorbed dyes. (**a**) Magnetic separation of MCZ particles from natural CZ powder by a permanent magnet; (**b**) magnetic agglomeration of SAP-MCZ (green) and RhB-MCZ (red) particles near the magnet. (**c**) Absorbance spectra of 5 mM SAP and supernatant of SAP-MCZ complex revealed ~ 70% adsorption of SAP by MCZ in PBS buffer solution.

**Figure 3:**
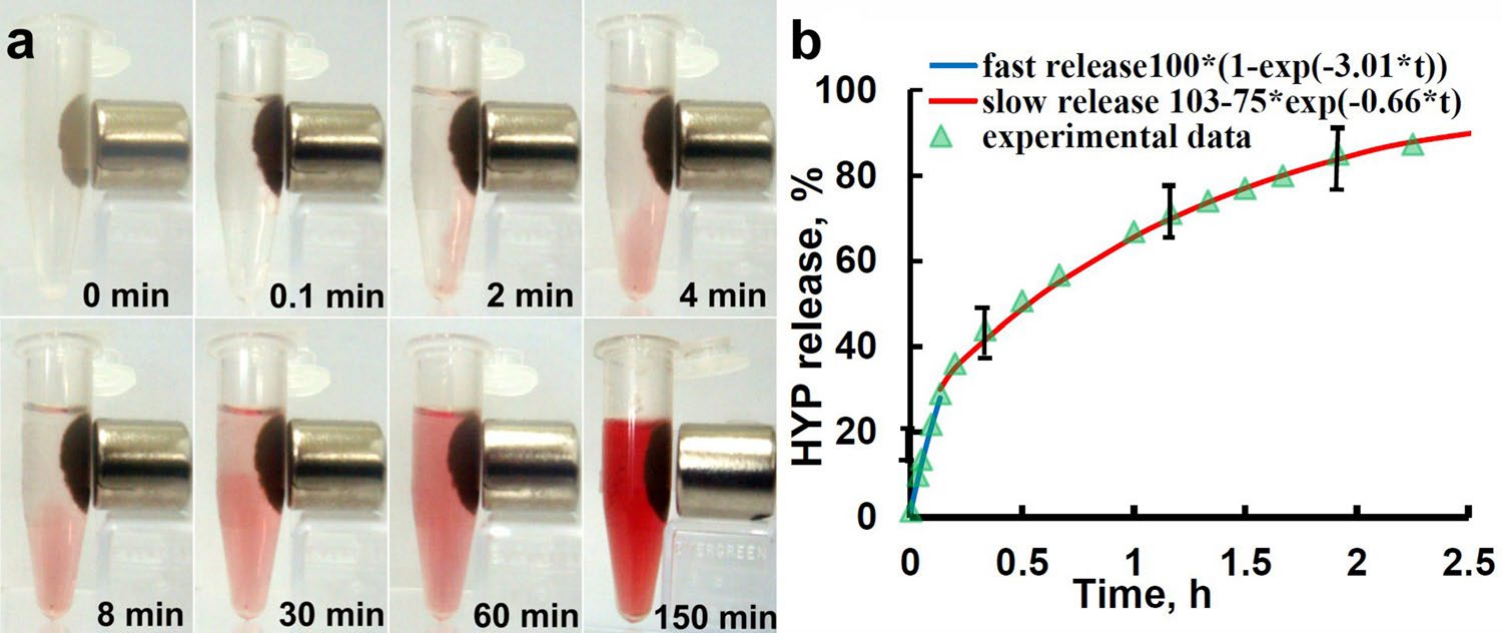
Time-dependent hypericin release from HYP-MCZ particles. (**a**) Photographs of continuos release of HYP molecules from HYP-MCZ micro-particles to ethanol–water 50% solution. (**b**) two-exponential kinetics of HYP release from MCZ pores measured by absorption spectroscopy.

Magnetic nano/micro-particles have been prepared by incorporation of iron oxide Fe_3_O_4_ (IO) nanoparticles into the CZ matrix to enhance their physical properties and to expand the area of bio-medical applications. The fabricated magnetic IO-CZ (MCZ) nano- and micro-particles have stable optical, magnetic and adsorption properties in dry condition (Fig. 2a), as well as in aqueous or ethanol-aqueous solutions. Furthermore, it is demonstrated that MCZ particles effectively adsorb sulfonated aluminum phthalocyanine (SAP), (Fig. 2b,c), rhodamine B (RhB) (Fig. 2b), hypericin (HYP) (Fig. 3) and other photosensitizers and fluorescence markers. As a temperature-dependent fluorescent dye, RhB can be used for measuring an alternating magnetic field-induced temperature changes in organs and cells in two-photon fluorescence microscopy.

Since HYP is poorly soluble in water^18^ and the absorbance of HYP in the aqueous solution is not proportional to the dye concentration, the release of HYP is measured in a 50% ethanol–water solution.

Figure 3a demonstrates HYP release within 2.5 h after adding of 50% ethanol–water solution to a dry HYP + MCZ system. The absorption of 5 µM HYP in 50% ethanol–water solution is considered to be 100%. The kinetics of HYP desorption is quantified by measuring of solution absorption at 592 nm at various times during the desorption process.

The rate of HYP release from MCZ pores can be characterized by two near-exponential curves (Fig. 3b). The first fast-phase continues from 0 to ~ 8 min and can be fitted by an exponential curve with the release rate constant k_1_ = 3.01 h^−1^ (blue line in Fig. 3b). The second slower-phase can be characterized by the rate constant k_2_ = 0.66 h^−1^ (red line in Fig. 3b). Two-phases desorption of HYP may be determined by different rates of HYP release from the surface and from the interior of the MCZ clusters near the magnet.

### Magnetic zeolite for hyperthermia

**Figure 4:**
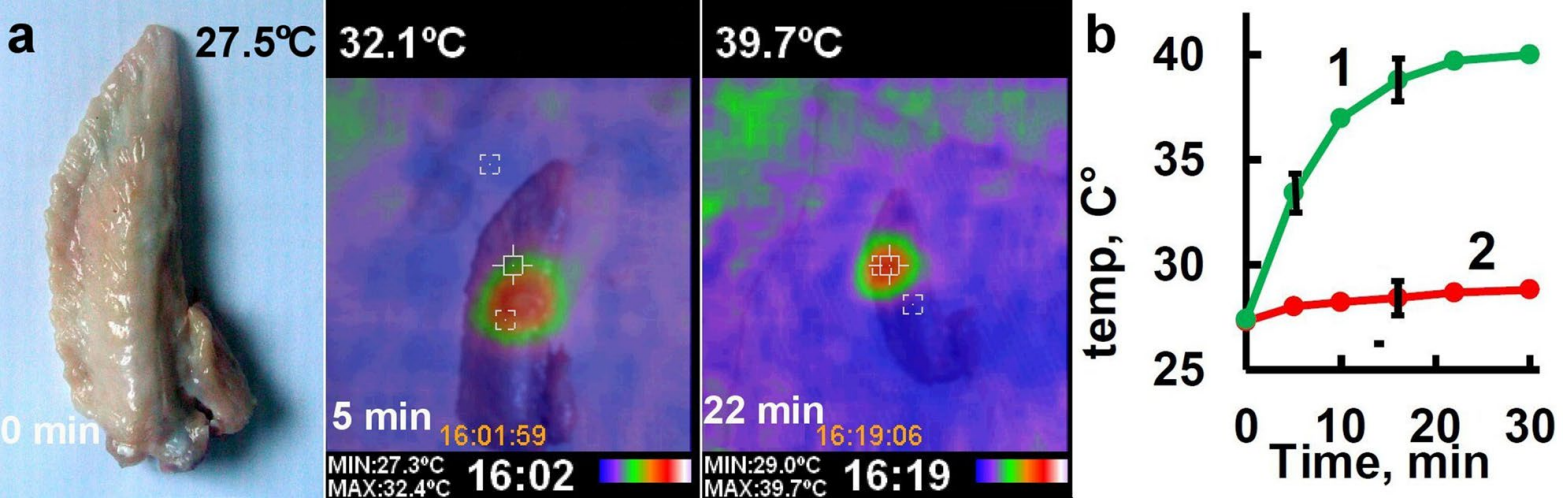
Alternating magnetic field induced local heating in chicken wing with injected MCZ nanoparticles (**a**) and monitoring of the temperature in the MCZ injection site (1) and the temperature at a distance of 2 cm from the site of injection in time (2) (**b**).

Hyperthermia at 39–45 °C has been shown to improve therapeutic outcomes in various tumors through its synergy with other therapeutic and diagnostic methods^2^. The development of multifunctional nanoparticles provides ample opportunities to integrate both monitoring and therapeutic modalities into a single effective cancer theranostic system. The fabricated MCZ particles in combination with a 300 W induction heater are used to induce a local hyperthermia in chicken organ in vitro. Chicken wings, freshly obtained from local markets, are used in this experiment. Before the MZC injection, the sample is soaked in 0.5% povidone-iodine solution for about 1 min and washed in phosphate-buffered saline. The temperature in the MCZ injected chicken wing is increased from 27.5 to 32.1 °C in 5 min and to 39.7 °C in 22 min under applying of alternating magnetic field Fig. 4. The dry weight of the utilized MCZ is 35 mg. The amount of MCZ can be significantly reduced by using a focused and more powerful alternating magnetic field in order to achieve a similar heating rate, allowing this approach to be applied in vivo.

### Assessment of anti-amyloidogenic activity of CZ particles

**Figure 5:**
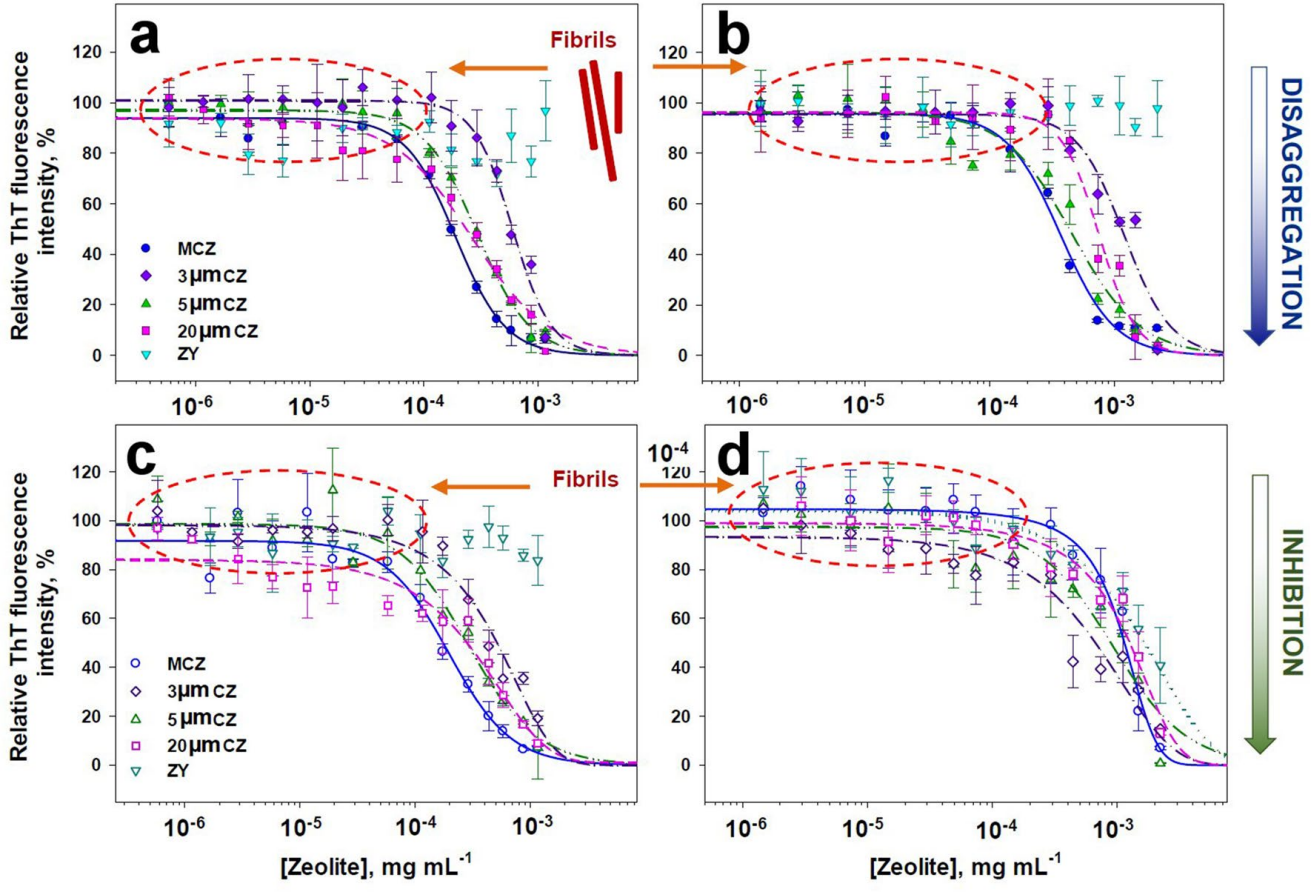
Quantification of CZ’s anti-amyloidogenic activity. Determination of the half-maximal disassembling concentrations of CZ particles for insulin (**a**) and lysozyme (**b**) and half-maximal inhibiting concentration for insulin (**c**) and lysozyme (**d**) quantified using ThT fluorescence assay. ThT fluorescence intensities of amyloid fibrils incubated in the presence of CZ particles (**a**,**b**) and amyloid fibrils formed in presence of CZ particles (**c**,**d**) were normalized to fluorescence measured after incubation of studied protein amyloid fibrils in the absence of CZs (taken as 100%). Each experiment was performed in triplicates; error bars represent the average deviation for repeated measurements of three separate samples.

Proteins are important biological macromolecules that are fundamental to the proper functioning of cells and organisms; therefore, the impact of nanoparticles in living organisms at the protein level is a critical issue that is attracting increasing attention. It is generally accepted that all peptides and proteins could be transferred into amyloid state, if appropriate, typically denaturing condition are applied in experiments in vitro. Similar morphology of amyloid fibrils and comparable tinctorial and physicochemical properties allowed us to apply a common approach based on spectrofluorometry measurements to control the quantity of fibrils in samples^22,23^. For study the disassembly effect of CZ and MCZ particles, the amyloid fibrils formed by insulin and lysozyme were treated with different concentrations of particles in the range from 0.01:1 to 1:20 of protein to CZ particles (mg/mg) at 37 °C for 24 h and ThT fluorescence assay has been employed. The cationic benzothiazole dye ThT undergoes significant enhancement of quantum yield when bound to fibrils, but fluorescence intensity of ThT is not affected by the presence of the globular proteins in the native state, proteins in molten globule or unfolded state, or by amorphous aggregates of protein. The relative fluorescence intensity calculated as a difference between fluorescence intensity of ThT in the control samples (taken as 100%) and the fluorescence intensity of ThT in the presence of amyloid aggregates and CZ particles serves to examine the effectiveness of CZ particles to disassemble already formed fibrils (Fig. 5). The sigmoidal decline of fluorescence intensities after treatment with CZ particles allowed us to determine the half-maximal disassembling (DC_50_) concentrations of studied CZ particles (the DC_50_ concentration of CZs required to disassemble preformed fibrils by 50%). As documented on Fig. 5, the ability to destroy pre-formed fibrils was dose-dependent and DC_50_ values of CZ particles were found to be in sub-mg/mL concentrations range. The calculated apparent DC_50_ values are summarized in Table 1. To obtain more detailed information regarding anti-amyloidogenic activity of CZ particles, formation of amyloid fibrils in the presence and absence of CZ particles was assessed (inhibition effect). As documented on Fig. 5, except synthetic zeolite (ZY) particles, sigmoidal decline of relative fluorescence was observed. The apparent half-maximal inhibitory concentration, IC_50_ (the concentration of CZs required to inhibit fibril formation by 50%) were calculated to be in sub-mg/mL concentrations range. Intriguingly, the determined IC_50_ and DC_50_ values for insulin were one-half lower in comparison to those determined for lysozyme (the p-value was found to be 0.004). In addition, anti-amyloid activity of MCZ particles was noticeable higher than natural CZ. Moreover, it is important to be noted that synthetic zeolite Y particles (from Sigma) exhibit only negligible inhibiting and disassembly effect.

**Table 1:** Inhibition, IC_50_ and disassembly, DC_50_ values of CZ particles determined for insulin and lysozyme.

CZ particles	Insulin		Lysozyme	
	IC_50_ (mg mL^−1^)	DC_50_ (mg mL^−1^)	IC_50_ (mg mL^−1^)	DC_50_ (mg mL^−1^)
3 µm	0.48 ± 0.025	0.6 ± 0.022	1.23 ± 0.075	1.2 ± 0.090
5 µm	0.28 ± 0.020	0.28 ± 0.010	0.85 ± 0.060	0.45 ± 0.035
20 µm	0.215 ± 0.013	0.23 ± 0.013	0.72 ± 0.045	0.55 ± 0.035
30 µm ZY	NA	NA	NA	1.85 ± 0.1
MCZ	0.175 ± 0.008	0.18 ± 0.008	1.0 ± 0.090	0.35 ± 0.017

**Figure 6:**
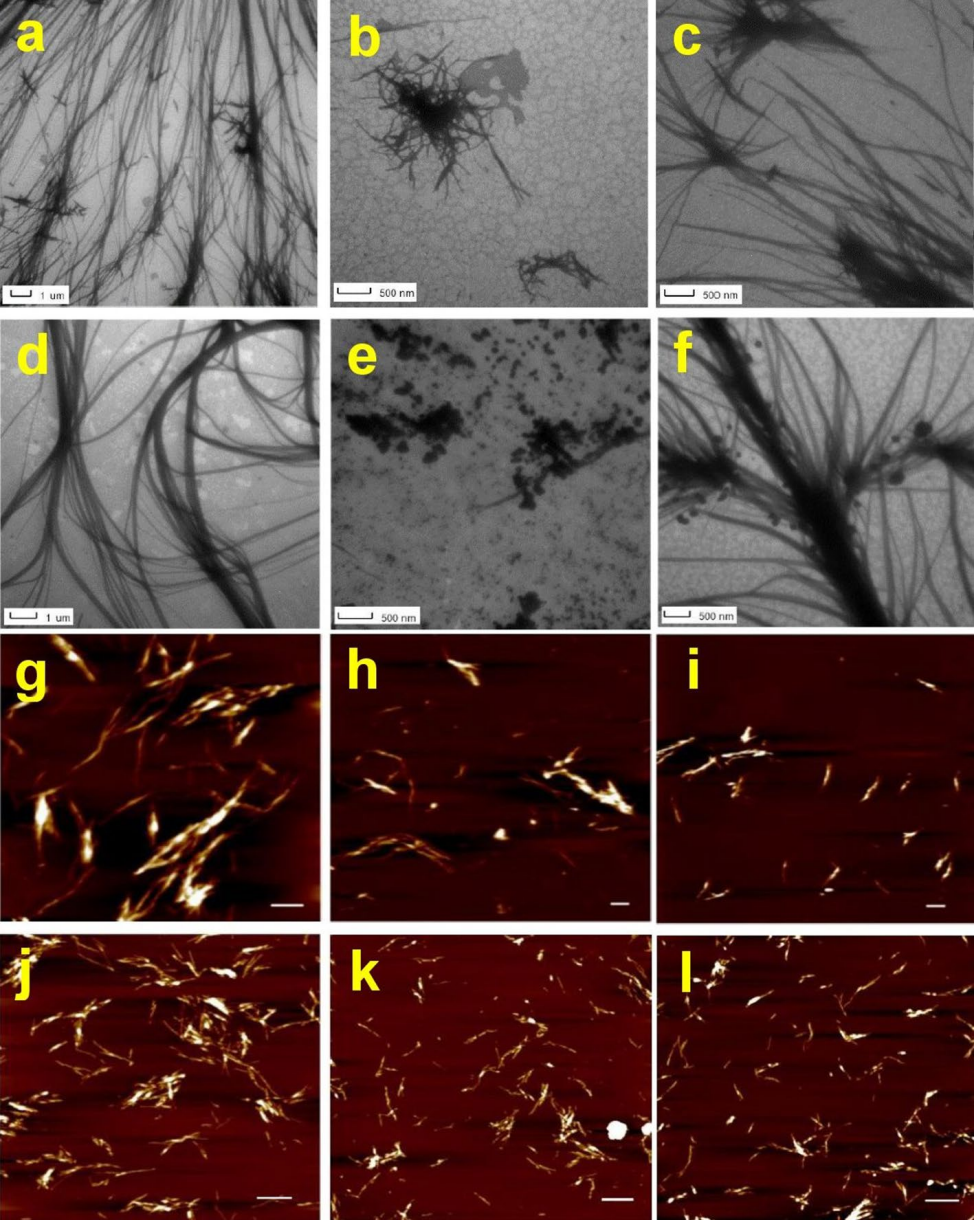
Microscopic determination of anti-amyloidogenic activity of CZ particles using TEM (**a**–**f**) and AFM (**g**–**l**) techniques. Disassembly effect of CZ particles was studied using lysozyme amyloid fibrils incubated alone (control) (**a**) and in the presence of MCZ (**b**), and 30 µm synthetic zeolite particles (**c**). Insulin amyloid fibrils alone (control) (**g**); incubated in the presence of 3 µm CZ (**h**) and 20 µm CZ particles (**i**) at the fibrils to nanoparticle concentration ratio of 1:5 (mg/mg). Inhibitory effect of CZ particles on insulin amyloid fibrils formed alone (**d**); in the presence of MCZ (**e**) and 30 µm synthetic zeolite particles (**f**). Lysozyme amyloid fibrils formed alone (control) (**j**) and in the presence of 3 µm CZ (**k**) and 20 µm CZ particles (**l**) at the fibrils to CZ particle concentration ratio of 1:5 (mg/mg). The data analyses were performed using NanoScope Analysis 1.20 software. White scale bars represent 1 μm.

Combination of transmission electron (TEM) and atomic force microscopy (AFM) has been used to confirm the results obtained by the ThT fluorescence assay, and to reveal the morphology of fibrils. The representative TEM and AFM images are presented in Fig. 6. The microscopic images of insulin and lysozyme amyloid fibrils (control), displayed the characteristic amyloid morphology. Several micrometers long fibrils and bundles were observed. However, when amyloid fibrils were incubated in the presence of studied CZ particles, the quantity of fibrils and their morphology were changed. As showed on Fig. 6b,c,h,i upon presence of CZ particles, reduced amount of typical fibrils and increased amount of shorter and thinner fibrils was detected. The most significant decrease of fibrils amount with dramatically changed morphology was observed in the presence of smaller particles and particles with incorporated magnetite (Fig. 6b,h). However, the incubation of synthetic zeolite particles with mature fibrils does not cause destruction of fibrils; only weak partial disaggregation of preformed fibrils was observed (Fig. 6c).

Similarly, the morphology and amount of fibrils were affected when amyloid fibrillization (inhibition study) occurred in the presence of CZ particles as documented on Fig. 6e,f for insulin, and on Fig. 6k,l for lysozyme amyloid fibrillization. Therefore, the microscopy techniques fully confirmed the results obtained by ThT fluorescence assay. The presence of natural CZ particles and magnetic CZ particles led to inhibition of fibrils formation and changing of their morphology, as documented by lower amount of thinner and shorter fibrillar structures. The addition of synthetic zeolite particles appears to be incapable to significantly affect fibril formation, as demonstrated on Fig. 6f.

**Figure 7:**
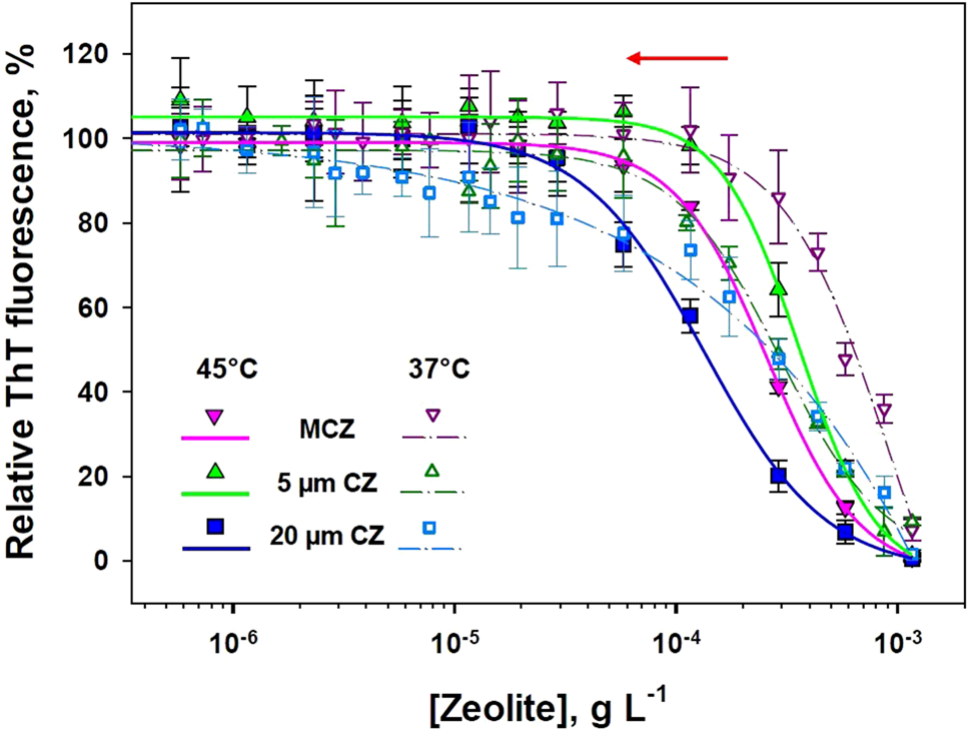
Determination of disassembly effect of CZ particles at normal and “hyperthermic” conditions monitored via ThT assay. Effect of selected CZ particles on amyloid fibrils was monitored at normal conditions (37 °C; open symbols and dash lines) and at “hyperthermic” conditions (45 °C; filled symbols and solid lines). The extent of destroying of amyloid fibrils was monitored using ThT fluorescence assay. The error bars represent the average deviation for repeated measurements of three separate samples.

In order to examine the “hyperthermic” anti-amyloidogenic effect of CZ particles, pre-formed fibrils were exposed to increased concentration of either, CZ or MCZ particles at 45 °C. After incubation the disassembly effect was analyzed using ThT fluorescence assay (Fig. 7). As it is shown on Fig. 7, at elevated temperature (45 °C), the disassembly effect of magnetite-loaded MCZ particles increased. Solid lines that fitted experimental data shifted left, to lower concentrations (as indicated by red arrow). The obtained results allowed us to assume that anti-amyloidogenic activity could be enhanced by co-application of external magnetic field and/or induction of hyperthermia. It should be noted however, that there is no shift for 5 µm and only small shift for 20 µm CZ particles was observed. These results were expected since CZ particles are not loaded with magnetite and therefore, did not response to the increased temperature.

## Discussion

Application of controlled drug delivery and the effect of drugs is limited by a variety of factors, including physicochemical properties of the drug, the rate of drug delivery, the route of administration and their inability to penetrate tissues due to their chemical structure as well as nature of carrier.

The IO-based NPs possess a unique property of superparamagnetism that confers advantages such as the generation of heat under alternating magnetic fields and the ability to be guided to a specific tissue or organ by an external magnetic field. Based on above mentioned advantages of mesoporous CZ particles we prepared multimodal composites containing photoactive dye and magnetic nanoparticles. Incorporation of magnetic nanoparticles into zeolite allowed to “direct” targeting using application of external magnetic field (Figs. 2, 3)^24,25^. We demonstrated the effective adsorption of RhB, SAP and HYP irrespective of incorporation of magnetite into CZ’s particles as showed in Figs. 2 and 3. Moreover, combination of discrete properties, such as non-toxicity, thermal stability, chemical tailorability, biocompatibility of CZ carriers, and adsorptions of different compounds allowed us to propose synergistic therapy approaches. Very recently, photothermal therapy using dual-functional nanoparticles (NPs) containing RhB was proposed by Wang et al.^26^. The authors suggested that the microscopic temperature detection can help to avoid overheating effects or insufficient heating, and thus can notably improve therapeutic efficacy, based on fluorescent temperature sensibility of RhB^27,28^. On the other hand, application of alternating magnetic field could induce temperature changes in tissues containing MCZ particles, resulted in significantly improved therapy effect with direct monitoring of temperature.

SAP is a well-studied photosensitizer which has been widely used in research and in clinical applications of the photodynamic cancers therapy^29^. Absorption spectrometry demonstrates up to 70% of adsorption efficiency of SAP in MCZ particles (Fig. 2). One of the best candidates for photodetection and photodynamic therapy is HYP due to its strong absorption spectrum with two major peaks at 545 and 590 nm. In this work, we have studied in detail the processes of adsorption and desorption by CZ particles. We demonstrate two-phase release of HYP from HYP-MCZ. Light absorption properties of HYP in ethanol–water environment allow to quantify the kinetics of desorption (Fig. 3) and two rate constants describing two-phase release of HYP from the surface (first fast-phase) and from the interior of MCZ (second slow-phase). Two-phase HYP release has been also detected from polycaprolactone-poly (ethylene glycol) (PCL-PEG) HYP loaded nanocapsules^30^. Previously, drug loading-dependent adsorption and release of HYP from nanoparticles has been demonstrated by Zeisser-Labouèbe et al.^31^. In addition, application of magnetic field via magnetic drug targeting combined with various treatment approaches, i.e. photodynamic therapy, hyperthermia can lead to preparation of more selective and effective treatment. In the present work prepared MCZ particles were tested for hyperthermia treatment. Application of alternating magnetic field resulted in tissue temperature increase of ~ 5 °C in the first 5 min and up to 39.7 °C in 22 min (Fig. 4). Therefore, drug targeting using external magnetic field may help to minimize side-effect and the effective adsorption of photodynamic dyes into cavities of MCZ particles provides more selective treatment.

One of the promising applications of CZ particles, is to detect, monitor and treat the amyloid-related diseases, which are characterized by the presence of protein amyloid aggregates. However, the evidence of the zeolite’s ability to affect protein amyloidogenesis is very limited^32^. Our task was to examine anti-amyloidogenic effect of different CZ particles. The experiments revealed that CZ particles, are capable to inhibit process of fibrils formation, as well as disaggregate preformed fibrils in nature-, size- and dose-dependent manner. It should be noted that synthetic zeolite Y did not exhibit ability to affect amyloid aggregation of insulin and lysozyme. Previously, Lucas and Keitz^32^ observed the effect of zeolites containing metal ions on the aggregation kinetics and the resulting structures of Aβ_1–40_ aggregates. In contrast to our data, the authors have found that synthetic zeolites strongly affect primary nucleation of Aβ_1–40_ in metal-ion presence dependent manner^32^. The discrepancy can be explained by two important differences in the experimental protocols: (i) Aβ protein is intrinsically disordered protein, therefore the effect of zeolites can be dissimilar, and (ii) in the cited work synthetic zeolites were loaded by metal ions^32^. Note that the chemical composition of the natural CZ, which we use includes (by weight %) CaO (5.48), K_2_O (2.22), Fe_2_O_3_ and FeO (1.79), MgO (1.28) and Na_2_O (0.79)^33^. Another suitable Alzheimer disease treatment using zeolite could be exploitation of anti-oxidative potential of zeolite as proposed by Montinaro et al.^16^. Application of microsized zeolite particles led to increased activity of the endogenous antioxidant enzyme superoxide dismutase (SOD) in the hippocampus of transgenic mice and a reduction of amyloid levels and plaque in treated transgenic mice^16^. A different approach using EMT-zeolite in preventing consequent cognitive damage due to abnormal Aβ–fibrinogen interaction showed that EMT zeolite NPs bind the fibrinogen molecules associated with the Aβ proteins, thus suppressing the Aβ–fibrinogen interaction within abnormal clots^34^.

Simultaneously with preparation of multimodal composites, we studied a possibility to enhance of anti-amyloid activity of CZ particles using “hyperthermia” approaches. Pre-formed fibrils were incubated not only at “normal” disaggregating condition (37 °C corresponding to normal human body temperature), but also at the “hyperthermic” condition, at 45 °C without application of magnetic field. Having demonstrated enhanced disassembly activity of selected CZ and magnetic CZ particles for amyloid fibrils by hyperthermia, we propose applicability of external magnetic field or photodynamic approaches for disassembly of amyloid structures. Moreover, incorporation of magnetic nanoparticles into CZ will improve drug delivery process, real-time monitoring of drug distribution surrounding a targeting side of tissue, as well as the subsequent effects of the therapeutics on the progression of diseases. In conclusion, magnetic zeolite particles have been fabricated to develop a new multifunctional magnetic and optical probe. The magnetic and anti-amyloidogenic properties have been demonstrated. We believe, that magnetic CZ nano/micro-particles can be considered as new multimodal probes for optical imaging and MRI, thermo- and phototherapy and as effective containers for controlled drug delivery. Further in vivo studies are needed to evaluate the efficacy of magnetic CZ nano/micro-particles in cancer hyperthermia therapy and destruction/inhibition of amyloid fibrils.

## Materials and methods

### Materials

Natural zeolite of clinoptilolite type (CZ) consisted of ~ 85% CZ was originated from Noyemberyan, Armenia. Synthetic zeolite Y microparticles (particle size ~ 30 µm, CAS No: 1318-02-1), magnetic iron oxide Fe_3_O_4_ (IO) nanoparticles (particle size 6.5 ± 3 nm, CAS No: 1317-61-9), Insulin, human recombinant, expressed in yeast (E.C. 234-279-7, I2643, ~ 24 IU/mg protein), Glycine (G7126), Lysozyme from hen egg white (E.C. number: 3.2.1.17, lyophilized powder, L 6876, ∼ 50,000 unit/mg protein), NaCl, Thioflavin T (ThT, T3516) were obtained from Sigma-Aldrich Corporation (St. Louis, MO; USA). Photodynamic dyes hypericin (HYP), and temperature sensitive dye rhodamine B (RhB) from Sigma-Aldrich were of analytical grade and used without further purification. Sulfonated aluminum phthalocyanine (SAP), a second-generation synthetic photosensitizer with a light absorption maximum at 675 nm was purchased from NIOPIC, Russia. All other chemicals were of analytical grade.

### Preparation of natural zeolite based magnetic composites

Natural clinoptilolite zeolite (CZ) mineral was ground to 30–50 µm by a mortar, purified by washing with distilled water using a fluidized bed process, and then dried at 105 °C. Nano/micro-sized CZ particles were obtained by grinding of minerals in a jet mill with subsequent sedimentation in aqueous solution. The sizes and shapes of particles were monitored by Scanning Electron Microscopy.

Magnetic CZ composites were prepared by adding CZ powder to the magnetic IO nanoparticles at IO/CZ weight to weight ratio of 1:3 and dried in an oven at 90 °C. Oleic acid was applied as surfactant for the IO stabilization. The obtained magnetic CZ composites (MCZ) were separated from nonmagnetic CZ by a simple magnetic procedure. The magnetism of MCZ samples was measured by a Lake Shore 7400 vibrating sample magnetometer at room temperature with the magnetic field of 20 kOe. The saturation magnetization of MCZ was about 3.7 A·m^2^/kg. Dye-MCZ composites were prepared by adding of dry MCZ nano- or micro-particles to solutions of a dye (HYP dissolved in ethanol, RhB in water, SAP dissolved in PBS buffer solution) and evenly dispersed with ultrasonic. Dye-MCZ composites were separated from the solution by centrifugation, transferred into air-tight containers and kept in the dark.

### Spectrometry and multiphoton imaging

Absorbance of dyes was measured by “SpectraSense” spectrometer (Princeton Instruments-Acton) with a spectral resolution of 1 nm.

The experiments were carried out by a similar method that was used to measure rates of HYP release from clinoptilolite zeolite in ethanol–water or in aqueous solutions of collagen, human hemoglobin, and other biomolecules^35^. Briefly, 5 mg of dry MCZ nanoparticles were added to 4 mL of 5 μM HYP solution in 50% ethanol–water and stirred. The absorption of this solution at 592 nm is considered to be 100%. Evaporation of ethanol from solution resulted in the adsorption of hydrophobic HYP on the MCZ particles. HYP/MCZ composites were separated from water using a magnet, a 4 mL of 50% ethanol–water solution is added to dry HYP/MCZ and the absorbance at 592 nm was measured over time.

For multiphoton microscopy, we used a system based on a laser scanning microscope LSM 510 META (Carl Zeiss, Jena, Germany) coupled to the Ti:Sapphire fs Tsunami laser^35^. Second harmonic generation imaging was performed using a Fluar water immersion 40×/1.2 NA, WD = 0.28 mm objective (Carl Zeiss, Germany) and the laser radiation with the following parameters: wavelength—780 nm, pulse width—120 fs, repetition rate—80 MHz, pulse energy—0.2 nJ.

### Anti-amyloidogenic activity assessment: ThT fluorescence analysis

For study the anti-amyloid activity of CZ particles, two different experimental approaches have been applied: (i) analysis of the amyloid fibrils formation in the presence of CZ particles (inhibition study); and (ii) amyloid fibrils disaggregation ability of CZ particles (disaggregation study). For inhibiting measurements, CZ particles in the range from 0.01:1 to 1:20 of protein to CZ particles (mg/mg) were added to freshly prepared protein solution (10 µM) in 70 mM glycine buffer containing 80 mM NaCl, pH 2.7. A mixture was incubated at 65 °C, 1200 rpm for 2 h. The anti-amyloid disassembling activity of CZ particles was investigated after the incubation of preformed amyloid aggregates (always 10 μM; fixed concentration) with CZ particles at the protein to nanoparticle ratio in the range from 0.01:1 to 1:20 of protein to CZ particles (mg/mg) at 37 °C for 24 h. The extent of the inhibiting and disassembling activity was detected by the Thioflavin T (ThT) fluorescence assay. ThT was added to each sample to reach a final ThT to protein molar ratio of 2:1. The fluorescence intensity was measured using a 96-well plate by a Synergy MX (BioTek) spectrofluorometer. The excitation was set at 440 nm and the emission was recorded at 485 nm. The excitation and emission slits were adjusted to 9.0/9.0 nm and the top probe vertical offset was 6 nm. Each experiment was performed in triplicates; all data represents a mean ± standard error of the mean of three independent experiments. Statistical analysis using Student’s t test was done with Sigma Plot 14.0 software (Systat Software Inc., IL, USA).

The IC_50_ (the concentration of CZs required to inhibit fibril formation by 50%) and DC_50_ (the concentration of CZs required to disassemble preformed fibrils by 50%) values were determined from fitting the dose-dependent experimental data using the nonlinear least-squares analysis with the sigmoidal sigmoid 3/4 parameter equation as described recently^36,37^.

### Anti-amyloidogenic activity assessment: microscopy techniques

Atomic force microscopy (AFM) imaging was performed on scanning probe microscope (Veeco di Innova, Bruker AXS Inc., Madison, WI) working in a tapping mode as described recently^38^. Briefly, the images were recorded using antimony (n) doped Si cantilever (NCHV, Bruker AXS Inc., Madison, WI) with spring constant 42 N m^−1^ and a resonance frequency of ~ 320 kHz. AFM images were acquired at a scan rates of 0.25 − 0.5 kHz with the resolution of 512 pixels per line (512 × 512 pixels/image) at 25 ± 1 °C. The images were examined using NanoScope Analysis 1.20 (Veeco di Innova, Bruker AXS Inc., Madison, WI, USA). We did not apply smoothing or noise reduction to obtained images. Samples for AFM were prepared by spotting of 5 μL of protein or protein fibrils (concentration was always 10 µM) on a freshly cleaved mica surface (highest grade V1 mica discs Ted Pella). After 5 min adsorption, the samples were washed with ultrapure water and gently dried under a soft stream of purified nitrogen gas.

Low voltage transmission electron microscopy (LVTEM) visualization of fibrils was performed using a Delong Instruments transmission electron microscope at an operating voltage of 5 kV. Samples were prepared by casting of aliquots on 400 mesh ultrathin carbon coated copper grids (Ted Pella) and dried.
